# Challenges in Identifying Biomarkers of Frailty Syndrome: A Systematic Review

**DOI:** 10.3390/medicina61071309

**Published:** 2025-07-21

**Authors:** Indira Omarova, Ainur Yeshmanova, Gulzhan Gabdulina, Aigul Tazhiyeva, Shynar Ryspekova, Akmaral Abdykulova, Ainur Nuftieva, Tamara Abdirova, Dame Sailanova, Zhanar Mombiyeva, Indira Karibayeva

**Affiliations:** 1 Department of General Medical Practice, Kazakh National Medical University Named After S.D. Asfendiyarov, 94 Tole bi, Almaty 050069, Kazakhstan; 2 Department of Normal physiology, Kazakh National Medical University Named After S.D. Asfendiyarov, 94 Tole bi, Almaty 050069, Kazakhstan; 3 Department of Clinical Disciplines, International Medical School, University of International Business Named After K. Sagadiev, Almaty 050069, Kazakhstan; 4 Department of Physical Medicine and Rehabilitation, Sports Medicine, Kazakh National Medical University Named after S.D. Asfendiyarov, 94 Tole bi, Almaty 050069, Kazakhstan; 5 Department of Health Policy and Community Health, Jiann-Ping Hsu College of Public Health, Georgia Southern University, Statesboro, GA 30460, USA

**Keywords:** frailty syndrome, biomarkers, gerontology, diagnostic, systematic review

## Abstract

*Background and Objectives*: The aim of this study is to categorize and combine (according to the source of biomaterial) biomarkers of frailty syndrome and identify challenges in research on these biomarkers by reviewing the current literature from the past five years. *Methods*: We systematically searching five electronic databases—PubMed, Scopus, Web of Science, CINAHL, and the Cochrane Library—for citations from 1 January 2019 to 1 July 2024. We conducted a qualitative data synthesis and categorized the limitations by topics and subtopics. PROSPERO—CRD: 42024491369. *Results*: A total of 61 papers met the criteria for inclusion in this study. These studies included a total of 56,758 participants, and 1479 unique biomarkers. We categorized biomarkers such as blood, genetic, urinary, and salivary biomarkers. Our analysis identified three major categories of challenges: challenges related to study design, unclear pathophysiological mechanisms, and biomarker-specific challenges. *Conclusions*: This review underscores the extensive research into biomarkers associated with frailty syndrome, such as blood, genetic, urinary, and salivary biomarkers. However, significant challenges persist, including methodological inconsistencies, biomarker measurement variability, and a limited understanding of underlying mechanisms.

## 1. Introduction

Frailty syndrome (FS) is a pathological, convertible aging process where frailty and comorbidity have a bilateral connectedness. In this review, we conducted a search for two main models of frailty, which were described earlier in the literature. The frailty phenotype describes frailty as a specific clinical condition characterized by the presence of at least three out of five criteria: reduced muscle strength, slow walking speed, low physical activity, feelings of fatigue, and unintentional weight loss [1]. The frailty index conceptualizes frailty as the accumulation of multiple health deficits detected through a comprehensive geriatric evaluation. Recent advances have deepened our understanding of the biological mechanisms underlying frailty [2]. The main concern is the potential for serious adverse outcomes after a minor stressor event or change. As life expectancy increases, a growing portion of the global population is reaching older age [3]. Initially, frailty was thought to revolve around three key physiological domains: neurological regulation, biomechanical function, and energy metabolism [4]. The proper recognition of FS and adequate treatment present a serious problem in geriatric assessment. According to the extensive literature on this topic, it is clear that FS is very complex, involving many areas and affecting many physiological systems [5].

Identifying FS in older populations has several advantages. In clinical settings, assessing frailty can support more informed treatment decisions and help predict outcomes in older adults, enabling more targeted and appropriate care [6,7]. The regular monitoring of frailty status also allows for the development of personalized intervention strategies, potentially slowing or even reversing frailty progression [8]. Furthermore, identifying frailty prevalence among older populations helps guide the planning, delivery, and assessment of community-based programs focused on prevention and management [9,10]. Research into FS has never been more prominent than it is now [11]. Progress regarding the identification, treatment, and prevention of FS has grown exponentially in recent years. Detecting signs of FS in the elderly has a number of advantages. In clinical practice, the assessment of the FS can be included in the decision-making process about treatment and predicting outcomes for the elderly and, in turn, determine the appropriate treatment [12]. Many studies have addressed the correlation of biomarkers and the FS clinical phenotype to a certain extent. Early diagnosis and timely intervention can slow or reverse aging and ensure the healthy aging of the elderly. In recent years, several markers, such as markers of nutrition, inflammation, and neuroimaging, have been associated with FS. However, a number of studies have examined the differences in such biomarkers in people who have demonstrated FS without comorbid diseases, and in people with FS caused by these diseases. Since chronic diseases often involve systemic inflammation, poor nutritional status, and neurodegenerative processes, it is likely that frail individuals with emerging health conditions accumulate detrimental biological changes linked to both aging and disease progression [13].

Currently, there are no gold standard biomarkers for diagnosing frailty. Frailty syndrome is increasingly recognized as a multi-dimensional construct, with candidate biomarkers falling broadly into three domains: clinical performance measures (e.g., gait speed, grip strength) that capture functional deficits [14], imaging biomarkers (e.g., muscle mass by DXA or MRI) [15] that quantify structural changes, and laboratory biomarkers (e.g., inflammatory cytokines, nutritional indices) that reflect underlying biological pathways [16]. In this review, we focus exclusively on laboratory biomarkers, both to leverage their potential for the early, subclinical detection of frailty processes (such as chronic inflammation and metabolic dysregulation) and because they offer objectively quantifiable, reproducible measures that can be standardized across settings. While clinical and imaging assessments remain indispensable for a comprehensive geriatric evaluation, laboratory assays may detect molecular perturbations before overt functional decline, thereby enabling timelier intervention. The aim of this study is to categorize and combine (according to the source of biomaterial) biomarkers of FS and identify challenges in research on these biomarkers by reviewing the current literature from the past five years.

## 2. Materials and Methods

### 2.1. Search Strategy

The PROSPERO database was searched on 22 April 2024 (PROSPERO—CRD: 42024491369), to identify registrations of comparable studies, but no similar study protocols were found. This systematic review was carried out with the support of a professional librarian. The review followed the PRISMA guidelines “http://www.prisma-statement.org/, accessed on 21 November 2023)” to ensure transparency and methodological rigor. We systematically queried PubMed, Scopus, Web of Science, CINAHL, and Cochrane Library. This review sought to provide a more contemporary account and included papers published between 1 January 2019 and 1 July 2024. The literature search was restricted to English-language publications and utilized only MeSH terms. We used the keywords “older adults”, “frailty syndrome”, “frailty”, “physical frailty”, and “biomarker”. The full literature search strategy can be found in Table S1.

### 2.2. Eligibility Criteria

Two independent reviewers screened the studies based on predefined eligibility criteria. Any disagreements between them were resolved through discussion and, if necessary, by consulting a third reviewer.

Inclusion criteria:

(1) Articles in English. (2) Studies with available full text. (3) Participants: Patients over 60 years of age. (4) Intervention: a biomarker for detection of FS is presented along with the challenges in biomarker research. (5) Outcome: the assessment of FS, reporting frailty with standard tools. All standardized tools were accepted [17], but the most common were as follows: Fried phenotype [1], Clinical Frailty Scale [18], Frailty Index of Accumulative Deficits [19], and FRAIL Scale [20].

Exclusion criteria:

Studies were excluded if one of the following criteria was met: (1) Duplicate publication. (2) Lack of access to essential data or full text, even after contacting the original authors. (3) Non-English language. (4) Review articles, meta-analyses, conference abstracts, retracted studies, commentaries, and editorials. (5) Title or abstract does not describe a relevant population, intervention, or outcome.

### 2.3. Data Extraction

Data extraction was performed by two reviewers using a Microsoft Excel spreadsheet. The following information was extracted: (1) characteristics of the study population (including sample size, demographics, country in which the study was performed); (2) setting in which the study was performed; (3) diagnostic criteria for FS; (4) measured biomarkers; (5) main associations reported; and (6) challenges of the study presented in the article.

### 2.4. Description of Analysis and Presentation of Data

For each included study, the following details were recorded: author, country, study design, setting, types of biomarkers investigated, sample size, participant age, type of analysis, key interpretations, and conclusions. A summary table was created to organize and present this information clearly. From the extracted data, we quantified the number of studies reporting on each biomarker. We divided the studies into four main groups of biomarkers: blood biomarkers, genetic biomarkers, biomarkers in urine, and biomarkers in saliva. Blood biomarkers were further subdivided as follows: inflammation biomarkers, protein biomarkers, vitamin biomarkers, lipid biomarkers, acid biomarkers, metal biomarkers, and enzyme biomarkers.

### 2.5. Qualitative Analysis

The thematic synthesis process consisted of three phases [21]. Using the qualitative analysis software Tableau Desktop 9.1 [22], we developed first level codes, and combined them to second level codes. Excerpts relevant to the focus of the systematic review were identified and assigned corresponding codes. The initial codes were subsequently grouped into categories and further developed (challenges of the study). From these categories, analytical themes were generated. To minimize researcher bias during the coding process and theme development, a collaborative approach was adopted to ensure objectivity and accuracy. Two independent researchers were involved in the coding process using Tableau software, allowing for the triangulation of the findings and resolution of any discrepancies through discussions and a second review. The sequence of themes is determined by the quantity of items assigned to each theme.

### 2.6. Risk of Bias (Quality) Assessment

The authors conducted a self-assessment of the risk of bias in a systematic review using the measurement tool for Evaluating Systematic Reviews (AMSTAR) [23]. AMSTAR is a critical assessment tool used to assess the methodological quality of systematic reviews. The updated version of AMSTAR-2 includes criteria for evaluating both randomized and non-randomized trials. AMSTAR-2 is used to identify potential errors or methodological deficiencies. The overall quality score is rated as low, moderate, or high.

## 3. Results

### 3.1. Data Search Results and Characteristics of Included Studies

**Figure 1:**
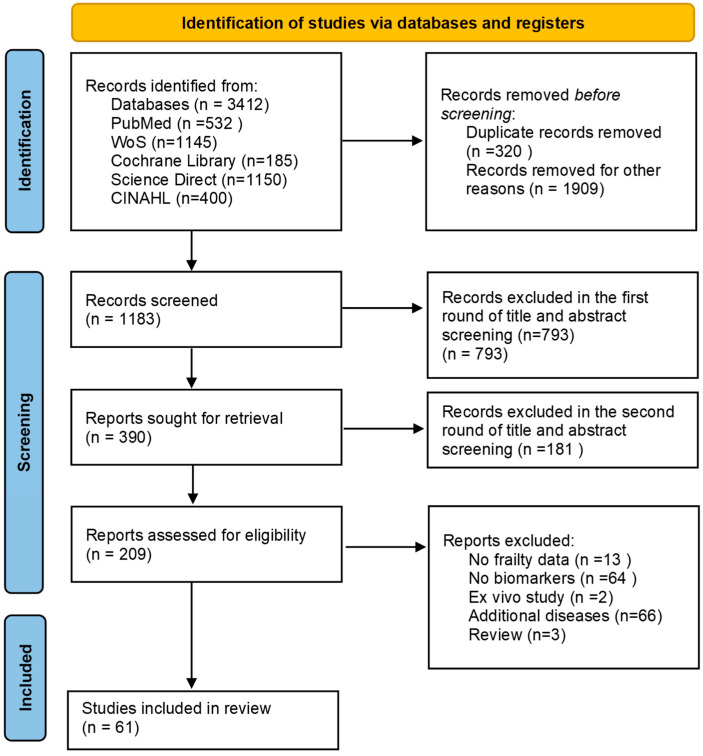
Study selection based on PRISMA methods.

Figure 1 presents the flowchart outlining the study selection process. The initial systematic search identified 3412 records. After removing duplicates and clearly irrelevant titles or abstracts, 1183 records remained for screening. Of these, 793 were excluded due to not meeting the inclusion criteria based on source relevance, leaving 390 articles for full-text review. Following this detailed assessment, 61 studies were deemed eligible for inclusion.

Detailed characteristics of the selected studies are provided in Table S2. Collectively, the studies involved 56,758 participants, with older adults defined as individuals aged 60 years and above. Various definitions of FS were used across studies, though the Fried phenotype was the most common and was applied in 41 studies (63%).

In total, 1479 unique biomarkers were studied. We divided the studies into four main groups of biomarkers: blood biomarkers (forty-six articles, 75%) [13,24,25,26,27,28,29,30,31,32,33,34,35,36,37,38,39,40,41,42,43,44,45,46,47,48,49,50,51,52,53,54,55,56,57,58,59,60,61,62,63,64,65,66,67,68], genetic biomarkers (eleven articles, 18%) [69,70,71,72,73,74,75,76,77,78,79] biomarkers in urine (two articles, 3%) [80,81], and biomarkers in saliva (two articles, 3%) [82,83]. Blood biomarkers were further subdivided as follows: inflammation biomarkers (seventeen articles), protein biomarkers (eleven articles), vitamin biomarkers (eleven articles), lipid biomarkers (two articles), acid biomarkers (2 articles), metal biomarkers (two articles), and enzyme biomarkers (one article).

#### 3.1.1. Challenges in FS Biomarker Research

##### Blood Biomarkers

For the biomarkers of inflammation, the major themes characterizing challenges in FS biomarker research were as follows: study design (seventeen articles), with subthemes including sample size (twelve articles), diagnosis (three articles), incomplete outcome data (two articles), experimental methods (three articles), confounders (five articles), study duration (four articles), sampling (six articles), unclear pathophysiological mechanisms (one article), with subthemes including insufficient evidence, and biomarkers (three articles), with subthemes including measurement and outcomes.

For the protein biomarkers, the major themes characterizing challenges in FS biomarker research were as follows: study design (nine articles), with subthemes including sample size, diagnosis, incomplete outcome data, confounders, and sampling; unclear pathophysiological mechanisms (three articles), with subthemes including insufficient evidence; and biomarkers (three articles), with subthemes including measurement and outcomes.

For the vitamin biomarkers, the major themes characterizing challenges in FS biomarker research were as follows: study design (eleven articles), with subthemes including sample size, study design, diagnosis, confounders, experimental methods, sampling, and study duration; unclear pathophysiological mechanisms (one article), with subthemes including insufficient evidence; and biomarkers (two articles), with subthemes including measurement.

For the lipid biomarkers, the major themes characterizing challenges in FS biomarker research were as follows: study design (two articles), with subthemes including sample size and confounders; and biomarkers (one article), with subthemes including measurement.

For the acid biomarkers, the major themes characterizing challenges in FS biomarker research were as follows: study design (two articles), with subthemes including study design and sampling.

For the metal biomarkers, the major themes characterizing challenges in FS biomarker research were as follows: study design (two articles), with subthemes including study design, confounders, and sampling; unclear pathophysiological mechanisms (two articles), with subthemes including insufficient evidence; and biomarkers (one article), with subthemes including measurement.

For the enzyme biomarkers, the major themes characterizing challenges in FS biomarker research were as follows: study design (one article), with subthemes including sample size, study design, confounders, and sampling. The challenges in FS blood biomarkers are presented in Table 1.

##### Genetic, Urine, and Saliva Biomarkers

For the genetic biomarkers, the major themes characterizing challenges in FS biomarker research were as follows: study design (ten articles), with subthemes including sample size, study design, diagnosis, sampling, and confounders; and biomarkers (three articles), with subthemes including measurement.

For the urinary biomarkers, the major themes characterizing challenges in FS biomarker research were as follows: study design (one article), with subthemes including study design, sampling, and diagnosis; and biomarkers (one article), with subthemes including measurement.

For the salivary biomarkers, the major themes characterizing challenges in FS biomarker research were as follows: study design (two articles), with subthemes including sample size, study design, experimental methods, and confounders; and biomarkers (two articles), with subthemes including measurement and correlation. The challenges in FS genetic, urine, and salivary biomarkers are presented in Table 2.

##### Risk of Bias (Quality) Assessment Results

According to the AMSTAR-2 assessment results presented in the Supplemental Materials (S3), the quality of the present systematic review is rated as moderate.

## 4. Discussion

This systematic review represents an effort to comprehensively categorize laboratory biomarkers associated with FS and to identify research challenges encountered over the past five years. Our findings offer a structured classification of laboratory biomarkers and highlight critical methodological and conceptual challenges in the field. We identified and categorized biomarkers into four primary groups: blood, genetic, urine, and saliva biomarkers. Blood biomarkers encompassed inflammation markers, proteins, vitamins, lipids, acids, metals, and enzymes. Inflammatory markers, notably interleukin-6 (IL-6), tumor necrosis factor-alpha (TNF-α), D-dimer, and fibrinogen, were the most frequently studied, underscoring the established link between chronic inflammation and FS. Protein biomarkers such as albumin, hemoglobin, and prealbumin were also prevalent, reflecting their association with nutritional status and muscle mass—key components of FS. Research in genetic biomarkers is expanding, with studies investigating polymorphisms in genes related to inflammation, oxidative stress, and muscle function, aiming to elucidate genetic predispositions to FS. Urine and saliva biomarkers remain underexplored, presenting potential avenues for future research to identify non-invasive indicators of FS.

The most promising biomarkers for clinical translation are accessible and modifiable indicators such as CRP [84], albumin, vitamin D, and IL-6 [30], as they reflect key pathophysiological processes (inflammation, nutrition, and endocrine status) and can be used for monitoring and intervention. At the same time, experimental and high-tech markers (GDF-15, myostatin, mitochondrial signals, genetic variants) have limited applicability and require further research and standardization before they enter routine practice [13,47,70].

The emphasis on inflammatory biomarkers aligns with earlier research highlighting the role of chronic inflammation in FS. Higher concentrations of pro-inflammatory cytokines, including interleukin-6 (IL-6) and tumor necrosis factor-alpha (TNF-α), have been repeatedly linked to a greater likelihood of developing FS [84]. Similarly, protein biomarkers, including albumin and prealbumin, have been linked to nutritional status and muscle mass, both critical components of FS [85]. The exploration of genetic biomarkers reflects a growing interest in understanding the genetic predisposition to FS, with studies investigating polymorphisms in genes related to inflammation, oxidative stress, and muscle function [86]. The identification of biomarkers using omics-based approaches helps to investigate the physiological mechanisms underlying FS and helps to assess the risk of developing and progressing frailty [87]. Different epigenetic biomarkers of frailty, from the first generation to the more specific and recent second-generation epigenetic aging biomarkers, may account for factors linked to different cellular types, such as heterogeneity, and a reverse causation process that requires integration with gene expression [88]. However, the limited research on urinary and salivary biomarkers suggests these areas remain underexplored, indicating potential avenues for future investigation.

Our analysis identified three major categories of challenges: challenges related to study design, unclear pathophysiological mechanisms, and biomarker-specific challenges.

Study design limitations: Many studies employed small sample sizes (less than 100) and cross-sectional designs, lacking longitudinal follow-up, which impedes the establishment of causal relationships. Heterogeneous populations and varying diagnostic criteria for FS further complicate comparability. Additionally, confounding factors such as comorbidities, medication use, and lifestyle influences were often inadequately controlled, despite their significant impact on biomarker levels and FS. Unclear pathophysiological mechanisms: The biological pathways linking specific biomarkers to FS are not well understood, hindering the development of targeted interventions and the translation of biomarker findings into clinical practice. Biomarker-specific issues: Inconsistencies in measurement techniques, timing of sample collection, and lack of standardized protocols contribute to variability in results. Single-time-point measurements may not accurately reflect the dynamic nature of biomarker levels associated with FS progression. It is possible that there may not be a single biomarker for FS. Many proposed biomarkers can be elevated in non-frail individuals due to other causes, limiting their specificity.

Our findings align with previous reviews that have highlighted the role of inflammatory markers in FS. For instance, a narrative review focusing on recent developments in FS research identified 22 articles on screening and diagnostic biomarkers, emphasizing the significance of inflammation markers [79]. Another review discussed mitochondrial biomarkers, though it identified only four relevant publications, indicating a nascent area of research [89]. Additionally, reviews have explored biomarkers involved in inflammation, mitochondrial dysfunction, neurodegeneration, and sarcopenia/osteoporosis, underscoring the multifaceted nature of FS [90]. Our systematic approach extends these findings by encompassing a broader range of biomarkers, including genetic, urinary, and salivary markers, and by providing a detailed assessment of research issues.

The challenges identified in our study regarding biomarkers of FS research align closely with those highlighted by Gupta and colleagues in their 2014 publication on biomarker research challenges such as biological diversity, disease heterogeneity, and technical limitations [91].

Based on the analysis of the challenges identified in our study, the following steps could advance the field of FS biomarker research:

1. Developing standardized protocols for laboratory biomarker measurement and FS assessment to enhance comparability across studies.
2. Conducting large-scale, longitudinal studies to elucidate causal relationships and the temporal dynamics of biomarkers in FS development.
3. Incorporating multifactorial analyses that account for confounding variables and explore interactions between biomarkers, comorbidities, and lifestyle factors.
4. Exploring the biological pathways linking biomarkers to FS to inform targeted therapeutic strategies.
5. Including diverse ethnicities and considering various aspects of FS (psychological, social, biological, environmental factors) to enhance the generalizability of findings.

Beyond biological substrates, accumulating evidence links FS to the structure and quality of older adults’ social connections and loneliness. In community-dwelling cohorts, limited social ties and low network diversity have been independently associated with higher frailty prevalence and accelerated frailty progression. For instance, according to the English Longitudinal Study of Ageing, after adjusting for age, sex, baseline frailty, and other confounders, high loneliness predicted a greater risk of pre-frailty (RRR = 1.74; 95% CI: 1.29–2.34) and frailty (RRR = 1.85; 95% CI: 1.14–2.99) approximately four years later [92]. Similarly, a meta-analysis of three observational studies reported a significant association between isolation among elderly and frailty (pooled OR 1.88, 95% CI 1.60–2.20) [93]. Mechanistically, poor social integration may exacerbate FS via increased systemic inflammation [94,95], reduced physical activity, and diminished access to health-promoting resources [94]. Integrating standardized assessments of social networks—such as the Lubben Social Network Scale—alongside laboratory biomarkers could therefore yield a more holistic FS profile and identify psychosocial intervention targets [96].

An important consideration is whether identified biomarkers are amenable to intervention. Inflammatory markers like IL-6 and TNF-α have been shown to decrease with structured exercise programs and dietary modifications—interventions that also improve muscle strength and physical performance in older adults [97]. Nutritional biomarkers (e.g., albumin, prealbumin, vitamin D) can be corrected through supplementation and dietary counseling, potentially slowing frailty progression [98]. Conversely, genetic polymorphisms represent fixed predispositions and are not directly modifiable, although they may inform personalized risk stratification [99].

Finally, the practical implementation of biomarker assessment in routine care hinges on cost, insurance coverage, and early FS diagnosis. The quantification of cytokines (IL-6, TNF-α) typically requires specialized immunoassays (ELISA or multiplex platforms), with per-analyte costs ranging from USD 50–200 in research settings, often burdensome for healthcare systems and patients when not already indicated. Nutritional biomarkers (albumin, hemoglobin) incur minimal incremental cost, as they are part of standard metabolic panels, whereas genetic testing and proteomic assays remain costly and are rarely covered outside research or high-risk clinical indications. Biomarkers for early FS diagnosis significantly improve the accuracy, timeliness, and personalization of the diagnosis of FS. Their use allows intervention before the onset of functional disability, improving quality of life and reducing medical costs. Thus, prioritizing accessible and modifiable biomarkers—such as nutritional indices and routine inflammatory markers—enhances feasibility for large-scale frailty screening and monitoring [100].

While our review focuses on biomarkers associated with FS, it is equally important to understand how these measures evolve during healthy aging. Longitudinal cohort studies have demonstrated that pro-inflammatory cytokines such as IL-6 and CRP gradually rise with advancing age—even in the absence of overt pathology—reflecting a low-grade “inflammaging” process [101]. Similarly, nutritional biomarkers (albumin, prealbumin) and endocrine factors (e.g., DHEA-S) decline slowly across the lifespan in healthy individuals, correlating with reductions in muscle mass and bone density [102]. Characterizing these baseline trajectories allows us to distinguish between the physiological drift of aging and the accelerated biomarker shifts that herald FS. Mapping individual biomarker slopes—from the gradual change seen in healthy elders to the steeper declines in frail populations—could identify critical inflection points for preventive interventions and help tailor strategies to flatten the disability curve ethically and effectively.

This study has several limitations: This review included only articles published in English, which may have led to the omission of relevant research in other languages. Additionally, dependence on the published literature could introduce publication bias, as studies reporting significant results are more likely to appear in academic journals. The focus on the past five years may have excluded earlier foundational studies that could provide additional insights into FS biomarkers. The findings cannot apply to a diverse population. Although psychosocial factors such as social network characteristics are increasingly recognized as important determinants of frailty, the assessment of social networks and their relationship with FS was beyond the scope of our biomarker-focused review. Consequently, we were unable to integrate social connectivity data into our analysis. A quantitative analysis was not performed due to significant heterogeneity across studies in terms of biomarker types, measurement methods, populations, and outcome definitions. This variability limited the feasibility of pooling data. Additionally, our review did not directly compare the predictive performance or clinical utility across the different biomarker categories (blood vs. genetic vs. urinary vs. salivary). Such head-to-head comparisons will be essential in future research to determine which markers—or combination of markers—offer the greatest accuracy, feasibility, and cost-effectiveness in frailty detection and monitoring.

## 5. Conclusions

This review highlights the growing body of research on biomarkers linked to FS, including those found in blood, urine, saliva, and genetic material. Among these, inflammatory markers like IL-6 and CRP, along with nutritional indicators such as albumin and prealbumin, emerge as the most promising for real-world clinical use. They are relatively easy to measure, cost-effective, and strongly associated with frailty risk—making them practical tools for early detection and monitoring.

On the other hand, while genetic markers and advanced omics technologies offer valuable insights into the biology of frailty, they are not yet ready for routine clinical application due to high costs, complexity, and lack of standardization. Similarly, urinary and salivary biomarkers, though attractive for their non-invasive nature, are still under-researched and require more rigorous, long-term studies to establish their utility. To move the field forward, future research must focus on standardizing how biomarkers are measured and interpreted; prioritizing low-cost, modifiable markers for clinical use; and exploring how these biomarkers change during healthy aging to better predict the onset of frailty. By tackling these challenges, we can improve early detection and personalized interventions—ultimately supporting healthier aging and better outcomes for older adults.

## Figures and Tables

**Table 1:** Challenges in FS blood biomarkers (46 articles).

Challenge Category	Challenge Subcategory	Specific Challenges	Publication
**Biomarkers of inflammation**			
Study design	Sample size	Small sample size	Buondonno et al., 2023 [25] Chew et al., 2019 [47] Hammami et al., 2020 [58] Hammami et al., 2020 [65] Liu et al., 2024 [67] Pansarasa et al., 2022 [26] Samson et al., 2022 [27] Semmarath et al., 2019 [28] Xu et al., 2022 [32]
		loss of a part of the cohort	McKechnie et al., 2021 [68] Xu et al., 2022 [32]
		depletion of the sample	Welstead et al., 2020 [31]
	Study design	-	None of the studies identified this theme
	Diagnosis	Subjective self-report	McKechnie et al., 2021 [68]
		self-reported data	Semmarath et al., 2019 [28]
		only one scale of frailty	Teixeira-Gomes et al., 2021 [30] Xu et al., 2022 [32]
	Incomplete outcome data	Lack of follow-up	Buondonno et al., 2023 [25]
		samples from another trial	Castro-Herrera et al., 2021 [36]
	Experimental method	No power calculation	Castro-Herrera et al., 2021 [36]
		second kind of error	Chew et al., 2019 [47]
		*p*-value was not adjusted	van Sleen et al., 2023 [29]
	Confounders	Severity of concomitant disease was not taken into account	Castro-Herrera et al., 2021 [36] McKechnie et al., 2021 [68] Zhang et al., 2022 [33]
		effect of drugs on biomarkers was not taken into account	Castro-Herrera et al., 2021 [36] Welstead et al., 2020 [31]
	Study duration	Short follow-up	Hammami et al., 2020 [58] Hammami et al., 2020 [65] Hsu et al., 2019 [24] Zhang et al., 2022 [33]
	Sampling	Heterogeneous group	Chew et al., 2019 [47] Hammami et al., 2020 [58] Hammami et al., 2020 [65] Hsu et al., 2019 [24] van Sleen et al., 2023 [29] Xu et al., 2022 [32]
Unclear Pathophysiological Mechanism	Insufficient evidence	No cause-and-effect relationships	Buondonno et al., 2023 [25]
Biomarker	Measurement	Not checked entire sample	Castro-Herrera et al., 2021 [36]
		measured at one point in time	Zhang et al., 2022 [33]
	Outcomes	There is no effect on long-term adverse clinical outcomes	Liu et al., 2024 [67]
**Protein biomarkers**			
Study design	Sample size	Small sample size	Arauna et al., 2020 [34] Sanz et al., 2021 [41] Valentini et al., 2019 [44]
	Study design	Cross-sectional data do not allow to establish a causal relationship	Sanz et al., 2019 [40]
	Diagnosis	Retrospectively based on clinical files	Angioni et al., 2022 [13]
		only one scale of frailty	Li et al., 2021 [38]
		body composition was determined using bioelectric impedance	Sanz et al., 2019 [40]
	Incomplete outcome data	Samples from another trial	Angioni et al., 2022 [13]
		data were missing due to lack of response and mortality	Shardell et al., 2019 [43]
	Experimental method	-	None of the studies identified this theme.
	Confounders	The effect of drugs on biomarkers was not taken into account	Sanz et al., 2021 [41]
		no data on use of anti-inflammatory or steroid drugs	Kamper et al., 2024 [35]
		no information about possible dehydration or fluid overload	Kamper et al., 2024 [35]
		lack of information about the deterioration in cognitive function	Sanz et al., 2021 [41]
	Study duration	-	None of the studies identified this theme
	Sampling	Heterogeneous group	Landino et al., 2021 [37]
Unclear Pathophysiological Mechanism	Insufficient evidence	No cause-and-effect relationships	Kamper et al., 2024 [35] Li et al., 2021 [38] Roh et al., 2022 [39]
Biomarker	Measurement	Does not reflect all the proteins	Landino et al., 2021 [37]
		blood samples were taken late	Kamper et al., 2024 [35]
		measured once	Shardell et al., 2019 [43]
	Outcomes	High correlation with the aging	Kamper et al., 2024 [35]
**Vitamin biomarkers**			
Study design	Sample size	Small sample size	Ngestiningsih et al., 2021 [51] Rattray et al., 2019 [54] Pillatt et al., 2021 [52]
	Study design	Cross-sectional study	Malaguarnera et al., 2020 [50] Xiao et al., 2020 [56] Kochlik et al., 2019 [48]
	Diagnosis	Self-reported data	Pilleron et al., 2019 [53] Xiao et al., 2020 [56]
		pre-weak condition was not taken	Xiao et al., 2020 [56]
	Incomplete outcome data	-	None of the studies identified this theme
	Experimental method	Large confidence intervals of causal estimates	Rattray et al., 2019 [54]
	Confounders	No data on concomitant diseases or medication	Henning et al., 2023 [46] Pillatt et al., 2021 [52]
	Study duration	Short follow-up	Henning et al., 2023 [46]
	Sampling	Heterogeneous groups	Gomez-Cabrero et al., 2021 [45] Kochlik et al., 2019 [48]
		exclusion of the weakest participants	Machado-Fragua et al., 2020 [49]
Unclear Pathophysiological Mechanism	Insufficient evidence	No cause-and-effect relationships	Vaes et al., 2019 [55]
Biomarker	Measurement	Measured at one point in time	Machado-Fragua et al., 2020 [49]
		use only one biomarker of vitamin K	Machado-Fragua et al., 2020 [49]
		no measured carnitine levels	Malaguarnera et al., 2020 [50]
	Outcomes	-	None of the studies identified this theme
**Lipid biomarkers**			
Study design	Sample size	Small sample size	Arauna et al., 2021 [57]
	Study design	-	None of the studies identified this theme
	Diagnosis	-	None of the studies identified this theme
	Incomplete outcome data	-	None of the studies identified this theme
	Experimental method	-	None of the studies identified this theme
	Confounders	No data on acute infections	Yin et al., 2023 [59]
	Study duration	-	None of the studies identified this theme
	Sampling	-	None of the studies identified this theme
Unclear Pathophysiological Mechanism	Insufficient evidence	-	None of the studies identified this theme
Biomarker	Measurement	Biological markers were measured using various analyzers	Yin et al., 2023 [59]
		biochemical markers of bone have not been assessed	Yin et al., 2023 [59]
	Outcomes	-	None of the studies identified this theme
**Acid biomarkers**			
Study design	Sample size	-	None of the studies identified this theme
	Study design	Cross-sectional study	Jang et al., 2020 [61]
	Diagnosis	-	None of the studies identified this theme
	Incomplete outcome data	-	None of the studies identified this theme
	Experimental method	-	None of the studies identified this theme
	Confounders	-	None of the studies identified this theme
	Study duration	-	None of the studies identified this theme
	Sampling	The average age of the participants was considered relatively young	Jang et al., 2020 [61]
Unclear Pathophysiological Mechanism	Insufficient evidence	-	None of the studies identified this theme
Biomarker	Measurement	-	None of the studies identified this theme
	Outcomes	-	None of the studies identified this theme
**Metal biomarkers**			
Study design	Sample size	Small sample size	Zawadzki et al., 2021 [63]
	Study design	-	None of the studies identified this theme
	Diagnosis	-	None of the studies identified this theme
	Incomplete outcome data	-	None of the studies identified this theme
	Experimental method	-	None of the studies identified this theme
	Confounders	Characteristics of chronic diseases were not taken into account	Wei et al., 2022 [62]
		coexistence of acute inflammatory diseases	Zawadzki et al., 2021 [63]
	Study duration	-	None of the studies identified this theme
	Sampling	Only one location	Wei et al., 2022 [62]
Unclear Pathophysiological Mechanism	Insufficient evidence	No cause-and-effect relationships	Wei et al., 2022 [62] Zawadzki et al., 2021 [63]
Biomarker	Measurement	The lead content in hair and bones not measured	Wei et al., 2022 [62]
	Outcomes	-	None of the studies identified this theme
**Enzyme biomarkers**			
Study design	Sample size	-	None of the studies identified this theme
	Study design	-	None of the studies identified this theme
	Diagnosis	-	None of the studies identified this theme
	Incomplete outcome data	-	None of the studies identified this theme
	Experimental method	-	None of the studies identified this theme
	Confounders	The effect drugs on biomarkers was not taken into account	Sanz et al., 2022 [64]
	Study duration	-	None of the studies identified this theme
	Sampling	-	None of the studies identified this theme
Unclear Pathophysiological Mechanism	Insufficient evidence	-	None of the studies identified this theme
Biomarker	Measurement	-	None of the studies identified this theme
	Outcomes	-	None of the studies identified this theme

**Table 2:** Challenges in FS genetic, urine, and salivary biomarkers (15 articles).

Challenge Category	Challenge Subcategory	Specific Challenges	Publication
**Genetic biomarkers**			
Study design	Sample size	Small sample size	Carini et al., 2022 [70] Inglés et al., 2019 [73] Iparraguirre et al., 2023 [74] Lee et al., 2022 [76]
		loss of a part of the cohort	Selenius et al., 2024 [71]
		insufficient recruitment skills	Martínez-Ezquerro et al., 2019 [77]
	Study design	Cross-sectional study	Lee et al., 2022 [76]
	Diagnosis	High heterogeneity that characterizes the frail phenotype	Iparraguirre et al., 2023 [74]
	Incomplete outcome data	-	None of the studies identified this theme
	Experimental method	-	None of the studies identified this theme
	Confounders	The severity of concomitant disease and drugs was not taken into account	Agostini et al., 2023 [69] Grasselli et al., 2022 [72]
		results obtained are distorted by subclinical stages of dementia	Mourtzi et al., 2019 [78]
		genetic and environmental factors	Mourtzi et al., 2019 [78]
	Study duration	-	None of the studies identified this theme
	Sampling	Conducted in only one location	Juárez-Cedillo et al., 2019 [75] Selenius et al., 2024 [71]
Unclear Pathophysiological Mechanism	Insufficient evidence	-	None of the studies identified this theme.
Biomarker	Measurement	Measured at one point in time	Lee et al., 2022 [76]
		small RNA sequencing was performed on whole blood samples	Carini et al., 2022 [70]
		the allele variant of T is not presented	Rabaneda-Bueno et al., 2021 [79]
	Outcomes	-	None of the studies identified this theme
**Urine biomarkers**			
Study design	Sample size	-	None of the studies identified this theme
	Study design	Cross-sectional study	Liang et al., 2020 [81]
	Diagnosis	Only one scale of frailty	Liang et al., 2020 [81]
	Incomplete outcome data	-	None of the studies identified this theme
	Experimental method	-	None of the studies identified this theme
	Confounders	-	None of the studies identified this theme
	Study duration	-	None of the studies identified this theme
	Sampling	Not fully representative sample	Liang et al., 2020 [81]
Unclear Pathophysiological Mechanism	Insufficient evidence	-	None of the studies identified this theme
Biomarker	Measurement	Circadian variability of cytokines	Jiang et al., 2020 [80]
	Outcomes	-	None of the studies identified this theme
**Salivary biomarkers**			
Study design	Sample size	Small sample size	Furtado et al., 2020 [82] Gómez-Rubio et al., 2022 [83]
	Study design	Cross-sectional studies are limited in their ability to determine causal relationships	Furtado et al., 2020 [82] Gómez-Rubio et al., 2022 [83]
	Diagnosis	-	None of the studies identified this theme
	Incomplete outcome data	-	None of the studies identified this theme
	Experimental method	Large number of biomarkers adds complexity to analysis	Furtado et al., 2020 [82]
		Different biological materials (saliva vs. blood) introduced in the statistical model	Furtado et al., 2020 [82]
	Confounders	The influence of all confounding factors was not eliminated	Gómez-Rubio et al., 2022 [83]
	Study duration	-	None of the studies identified this theme
	Sampling	-	None of the studies identified this theme
Unclear Pathophysiological Mechanism	Insufficient evidence	-	None of the studies identified this theme
Biomarker	Measurement	Individual variability in biomarkers studied	Furtado et al., 2020 [82]
	Outcomes	Inconsistent findings on the link between salivary IL-6 and dental/periodontal diseases	Gómez-Rubio et al., 2022 [83]

## Data Availability

The data presented in this study are available in the article.

## Supplementary Materials

The following supporting information can be downloaded at: https://www.mdpi.com/article/10.3390/medicina61071309/s1, Table S1: Search strategy of the Systematic Review of the challenges in Identifying Biomarkers of Frailty Syndrome, Table S2: Articles characteristics of biomarkers, S3: AMSTAR-2 assessment results [103].

## Abbreviations

FS	Frailty syndrome
FI	Frailty index
CRP	C-reactive protein
IL-6	Interleukin 6
TNF-α	Tumor necrosis factor
RNA	Ribonucleic acid
DNA	Deoxyribonucleic acid
NLR	Neutrophil–lymphocyte ratio
GDF-15	Growth Differentiation Factor-15
