# Supplementary material for: Challenges in Identifying Biomarkers of Frailty Syndrome: A Systematic Review
Source: Medicina (Kaunas). 2025 Jul 21;61(7):1309. doi: 10.3390/medicina61071309 (PMC12298523; doi:10.3390/medicina61071309)
Supplement: Supplementary file 1 [file medicina-61-01309-s001.zip › medicina-3734743-supplementary-final.pdf]

S1 Table. Search strategy of the Systematic Review of the challenges in Identifying Biomarkers of Frailty Syndrome.

**PubMed**	**Scopus**	**Web of Science**	**Cochrane Library**	**CINAHL**
#1 (aged) OR ("aged, 80 and over") OR (aging) OR (older) OR (elder) OR ("older adults") OR ("oldest old") OR ("very old") OR ("very elderly")<br>#2 ("frail elderly") OR ("frailty elderly") OR ("frailty index") OR ("frailty syndrome") OR ("physical frailty") OR ("frail scale") OR (fragility) OR ("pre-frailty") OR ("frailness")<br>#3 (biomarker*) OR (serum markers) OR (metabolic markers) OR (hormonal*) OR (hormonal markers) OR (inflammatory markers) OR (stem-cell changes) OR (metabolites) OR (blood-based biomarkers) OR (plasma biomarker)<br>#4 (validation) OR ("predictive value") OR (sensitivity) OR (evaluation) OR (validity) OR (accuracy)<br>#5 #1 AND #2 AND #3 AND #4<br>#6 ("Systematic review"[Publication Type] OR "review"[Publication Type] OR "meta-analysis"[Publication Type] OR "scoping review"[Title/Abstract])<br>#7 #5 NOT #6<br>#8 English[Language]<br>#9 #7 AND #8	Criteria: Search in All Fields.<br>Filter: include only Content type: Research Articles.<br>Search date: January 2019 - July 2024<br>1. Frailty syndrome AND Biomarkers: search in Title/ abstract or Keywords	Criteria: Search in All Fields.<br>Filter: include only "Articles"<br>Search date: January 2019- July 2024<br>Topic:<br>1. Frailty<br>2. Biomarkers<br>Keyword:<br>1.Frailty syndrome<br>2.Biomarkers of aging	Criteria: Search in All Fields.<br>Filter: include only "Articles"<br>Search date: January 2019- July 2024<br>1. Biomarkers of frailty: search in Title/ abstract or Keywords	Criteria: Search in All Fields.<br>Filter: include only "Articles"<br>Search date: January 2019- July 2024<br>1. Frailty syndrome

#10 (“2019/01/01”[Date - Publication] : “2024/07/01”[Date - Publication])<br>#11 #9 AND #10				

S2 Table. Articles characteristics of biomarkers (61 articles)

**Blood biomarkers: Biomarkers of inflammation**								
**Publication**			**Study population**			**Biomarkers**	**Results**	**Limitations**
**Author**	**Country**	**Sample size**	**Age, yr (mean)**	**Frailty definition**	**Type of population**			
Buondonno et al.,2023[25]	Italy	20	82.0	Fried criteria	case–control study	CD4+ and CD8+ T cells, cytokine	The number of CD4+ and CD8+ T cells was reduced in the elderly	small sample size and lack of follow-up; the lack of a clear causal mechanism linking weakened immunity and mitochondrial dysfunction with frailty
Castro-Herrera et al., 2021[36]	United Kingdom	184	85.3	Frailty index	cross-sectional study	CRP,IL-1ra, soluble E-selectin,IP-10,sVCAM-1, IP-10, TNFRII	The relationship between age-related frailty and inflammation, as well as the correlation between increased age and inflammation in older individuals, is not mutually exclusive.	not all immune indicators were available to all 184 participants; samples were taken from participants in a randomized controlled trial and this required the exclusion of some residents of the nursing home; data on concomitant diseases or medication were not collected; no power calculation was performed
Chew et al., 2019[47]	Singapore	200	67.9	modified Fried criteria	Cross-sectional study	serum myostatin, insulin-like growth factor 1, total white cell count, CRP, IL-6 and TNFaR1	The serum myostatin level, adjusted for TBLM in males and IGF-1 in females, could serve as a potential indicator for frail individuals with a low relative appendicular skeletal muscle mass.	The study's design, which involves comparing different groups, does not permit the identification of a cause-and-effect connection between medical features, biomarkers, sarcopenia, and frailty; the sample size does not allow for a more detailed analysis of subgroups, and a second kind of error cannot be excluded;The participants in this study were chosen based on their health and cognitive abilities, which may limit the generalizability of the findings. The results pertain to a broader group of elderly individuals with health issues.
Hammami et al., 2020[58]	Tunis	141	77.0	Short Emergency Geriatric Assessment (SEGAm)	cross-sectional study	TNF-α, IL-8, IL-6, CRP	The state of frailty was linked to increased inflammation, as those who were frail had significantly higher levels of C-reactive protein, tumor necrosis factor alpha, and interleukin-8 in their blood.	The study was conducted on a small group of participants, and the observation period was relatively short. Moreover, many of the participants were already in a critical state.
Hammami et al., 2020[65]	Belgium	124	85.9	Short Emergency Geriatric Assessment (SEGA)	retrospective cohort study	C-reactive protein, full blood count, blood creatinine, vitamin D, and serum protein electrophoresis.	SEGA score was negatively correlated to albumin levels and positively correlated to CRP	small group of participants, and the observation period was relatively short, many of the participants were already in a critical state.

Hong et al., 2019[66]	China	380	86.8	frailty phenotype	cross-sectional study	TNF, prealbumin, total protein, albumin, retinol-binding protein, hemoglobin	Patients who were admitted to the hospital and had better nutritional status, as well as higher levels of TNF, TP, and ALB, were less likely to experience frailty.	This study is cross-sectional and does not confirm the conclusions of causation; the participants were from one hospital, not from several centers
Hsu et al., 2019[24]	Australia	1705	81.3	Fried frailty phenotype and Rockwood frailty index	cross-sectional study	Twenty-seven inflammatory biomarkers	IL-6 and IL-8 may be linked to frailty, but it is not clear whether they are the direct cause of frailty.	relatively short follow—up period of 3 years; the study was limited to men living in communities, and therefore may not be applicable to women, as well as to non-European peoples who were not widely represented in the study; CRP was not measured
Liu et al., 2024[67]	China	53	72.1	FRAIL scale	prospective cohort study	Leptin, irisin, decorin, and myostatin.	The release of inflammatory myokines, such as leptin, myostatin, can have a detrimental effect on muscle mass and strength in older individuals.	there is no confirmation of the effect of biomarkers on long-term adverse clinical outcomes; small sample size
McKechnie et al., 2021[68]	UK	1225	79.8	Fried phenotype	prospective study	Highly sensitive cardiac troponin T, N-terminal pro B-type natriuretic peptide, C-reactive protein, interleukin-6.	IL-6, hs-cTnT is associated with incident frailty	the severity of each concomitant disease was not taken into account; part of the initial assessment and the entire subsequent indicator are based on subjective self-report; The group comprises solely males and is predominantly white, with European ancestry, which may restrict the applicability of the findings. A fifth of the initial group was not followed up.
Pansarasa et al., 2022[26]	Italy	219	76.7	Frailty Index	longitudinal population-based study	CRP and pro- and anti-inflammatory cytokines	A substantial rise in the levels of C-reactive protein, interleukin-1β and -6, and tumor necrosis factor alpha in the bloodstream of frail individuals.	the sample size was not large;
Samson et al., 2022[27]	Netherlands	34	69.4	frailty index	Cohort Study	STAT1,3,5 phosphorylation in monocytes, B cells, CD4+, CD8+ T cells upon stimulation with IL-2, IL-6, IL-10, IFNα and IFNγ	The findings underscore the significance of intact JAK-STAT signaling in the process of healthy aging and suggest that cellular pSTAT levels could serve as a potential biomarker for frailty.	The number of participants in our study was limited, which means the findings cannot be applied to the broader population.
Semmarath et al., 2019[28]	Thailand	526	68.6	Fried frailty phenotype	cross-sectional study	C-reactive protein (CRP), interleukin-6 (IL-6), insulin-like growth factor-1, CD4+:CD8+	The concentrations of IL-6 and CRP in the blood of frail individuals were notably higher than those in non-frail individuals, and these levels were strongly correlated with the frailty status.	The sample size of the elderly population and the incorporation of certain self-reported information, which could impact the outcomes.
Sleen et al., 2023[29]	Netherlands	317	65.0	Frailty Index	prospective cohort study	29 circulating markers of inflammageing	The levels of IL-6, C-reactive protein, IL-1 receptor antagonist, YKL-40, and elastase were linked to frailty.	The male participants in this research were, on average, more mature than the female participants; the FI was not used due to logistical constraints; the p-value was not adjusted
Teixeira-Gomes et al., 2021[30]	Portugal	291	83.6	comprehensive geriatric evaluation, Fried frailty phenotype	Cohort Study	vitamins A, E and CRP, IL-6, oxidative DNA levels	IL-6, neopterin, and Kyn/Trp have shown promise as potential predictive biomarkers for frailty syndrome.	No notable variations or correlations were observed for oxidative stress markers, indicating that the data does not support the oxi-inflamm-aging hypothesis.<br>The study did not consider the multifaceted nature of frailty, which encompasses psychological, social, biological, and environmental aspects.

Welstead et al., 2020[31]	Scotland	3204	69.0	Frailty Index, Fried phenotype	Longitudinal Cohort Study	Fibrinogen and C-reactive protein	The research revealed a consistent correlation between elevated CRP and Fibrinogen levels in the first wave and the progression of frailty, as measured by the Frailty Index, but not the Fried Phenotype.	calculated transitions over a 12-year period when there was a depletion of the sample; did not take into account anti-inflammatory drugs as a covariate that could affect the results
Xu et al., 2022[32]	China	3267	87.0	38-item frailty index	Longitudinal cross-sectional Study	neutrophil-lymphocyte ratio	Elevated neutrophil-to-lymphocyte ratios were linked to a greater likelihood of frailty, both existing and developing.	Only use FI to determine the status of fragility; The sample size for analysis was relatively small due to attrition and mortality. The study was conducted among elderly individuals residing in the community, and the findings may not be applicable to elderly patients, particularly those with inflammatory conditions. There may still be residual confounding factors influencing fragility.
Zhang et al., 2022[33]	China	1822	78.0	Fried phenotype	Longitudinal observational, prospective and community-based cohort study	neutrophil-to-lymphocyte ratio (NLR), platelet-to-lymphocyte ratio (PLR) and systemic immune-inflammation index (SII)	NLR and SII are readily available indicators of immunity that can be utilized to anticipate the onset of frailty in clinical settings.	The immune markers were assessed at a single point in time, without considering any subsequent temporal variations. Additionally, there was no evaluation of immune cell functionality or systemic inflammation.
**Protein biomarkers**								
Angioni et al., 2022[13]	France	1199	77.5	Fried criteria	case-control study	GDF15, TNFR1, omega 3 index	Rising trends in inflammatory indicators and declining trends in nutritional indicators.	this work is a secondary analysis that was not specifically designed to measure biomarkers of fragility; the classification of subtypes of frailty was performed retrospectively based on clinical files
Arauna et al., 2020[34]	Chile	55	74.0	Fried's Phenotype	prevalent-case control study	Platelet aggregation, activation plasma levels of Thromboxane B2, 8-isoprostane, Growth Differentiation Factor-15	The heightened oxidative stress and elevated GDF-15 levels may be linked to changes in platelet reactivity in elderly individuals.	The study did not examine experiments with sub-acute doses of agonists.<br>A major issue for these potential biomarkers is their strong association with age, making it challenging to distinguish the impact of frailty.
Kamper et al., 2024[35]	Denmark	1036	78.9	Clinical Frailty Scale	cross-sectional study	Growth differentiation factor-15 (GDF-15)	The ideal threshold for GDF-15, in relation to the existence of sarcopenia and frailty.	systemically measured aging may not reflect aging acting at the local cellular level; the relative contribution of muscle tissue to overall systemic aging may differ from the contribution of other tissues; blood samples were taken within 24 hours after acute hospitalization; the potential effect of acute disease was controlled by adjusting CRP levels in statistical analyses; no data on early the use of anti-inflammatory or steroid drugs upon arrival at the emergency room, which could affect the concentration of biomarkers; no information about possible dehydration or fluid overload; impossible to establish cause-and-effect relationships
Landino et al., 2021[37]	Italy	752	73.6	Fried's frailty criteria	community-based cohort study	1301 proteins proteomic profile	The presence of creatine kinase M-type, B-type, C-X-C motif chemokine ligand 13, and thrombospondin 2 was linked to a state of frailty.	1,301 proteins were measured using atomic analysis, which does not reflect all the proteins contained in plasma; the number of volunteers with impaired health in this group was small

Li et al., 2021[38]	China	619	69.3	Fried phenotype	cross-sectional study	Hemoglobin	Women admitted to the hospital with lower levels of hemoglobin were more likely to experience frailty.	The information was gathered in a comprehensive tertiary healthcare facility, and therefore, the findings cannot be generalized to all sectors of the medical industry; the state of frailty was assessed by the phenotype of FS; the study was transverse, which does not allow conclusions to be drawn about the causal relationship between the identified associations
Roh et al., 2022[39]	South Korea	302	75.0	Fried phenotype	Cohort Study	Brain-derived neurotrophic factor (BDNF)	The concentration of BDNF in the plasma was found to be strongly linked to frailty in elderly individuals living in the community.	the presence of a causal relationship has not been determined; study was limited to Korean men and women
Sanz et al., 2019[40]	Spain	112	84.9	Fried Frailty Criteria,Clinical Frailty Scale and the Tilburg Frailty Indicator	RCT	adiponectin	Adiponectin is associated with body composition, cognitive performance, and anxiety in long-term care facility residents, with varying relationships depending on gender.	Due to the nature of this study, it is not possible to infer a cause-and-effect relationship based on the findings. Furthermore, the body composition was assessed using bioelectrical impedance analysis rather than computed tomography, magnetic resonance imaging, or dual-energy X-ray absorptiometry.
Sanz et al., 2021[41]	Spain	103	84.7	Fried Frailty Criteria, the Clinical Frailty Scale and the Tilburg Frailty Indicator	RCT	Serum alpha-klotho (s-klotho) protein	Low s-klotho levels were linked to a greater likelihood of frailty. Furthermore, individuals with lower s-klotho levels experienced more falls in the six months following the initial evaluation.	The study had a limited sample size, insufficient data on the medications used by the participants, and potential changes in cognitive abilities over the six-month follow-up period that could impact the analysis.
Sanz et al., 2022[42]	Spain	225	84.8	ADL,Fried Frailty Index, Tilburg Frailty Indicator, Clinical Frailty Scale	RCT	serum sestrin-1	A higher level of sestrin-1 in the blood was linked to higher scores on the frailty scale, greater reliance on others, and diminished physical abilities and activity.	The research was carried out on a group of individuals residing in care facilities. Consequently, the findings cannot be generalized to other populations that do not meet the requirements for participation in this study.
Shardell et al., 2019[43]	Italy	774	74.6	Fried frailty phenotype	prospective cohort study	hormone klotho	Elevated levels of plasma klotho were linked to a reduced risk of frailty, particularly exhaustion.	biomarker concentrations were measured once, possibly with an error; data were missing due to lack of response and mortality; the possibility of unmeasured factors was not excluded
Valentini et al., 2019[44]	Italy	172	79.6	SHARE-FI	observational study	osteoprotegerin	Its potential as a biomarker for the geriatric frailty syndrome.	The sample was not extensive.
**Vitamin biomarkers**								
Gomez-Cabrero et al., 2021[45]	France Spain Italy	1522	75.3	Fried criteria	case control	vitamin D3,lutein zeaxanthin,miRNA125b-5p, cardiac troponin T, miR125b-5p, pro-BNP, sRAGE	oxidative stress, vitamin D demonstrated a statistical association with frailty	heterogeneous cohort of participants
Henning et al., 2023[46]	Spain	1271	75.6	Fried criteria	cross-sectional study	α-carotene, β-carotene, lycopene, lutein, zeaxanthin, β-cryptoxanthin, α-tocopherol, γ-tocopherol, retinol	The biomarkers of vitamin E, A, and lycopene were found to be elevated, while those of carotenoids were reduced, in individuals with frailty.	in most cohorts, there was no data on the status of body weakening for five years, which did not allow us to assess the role of dietary biomarkers in the development of body weakening; additional variables, such as medication intake, were not included in the cross-sectional analysis

Kochlik et al., 2019[48]	France Spain Italy	1450	81.4	Fried's frailty criteria	Cohort study	vitamins A, D3 , E, α-carotene, β-carotene	Both low levels of FMN and high levels of PrCarb are linked to pre-frailty and frailty.	this study was cross-sectional, it is not possible to conclude what leads to a FS of the body. The information regarding socioeconomic status and earnings was not provided, and thus it is not incorporated into the models with multiple variables.
Machado-Fragua et al., 2020[49]	Netherlands	644	60	Frailty Index	Prospective cohort study	vitamin K	The initial level of vitamin K in the blood was found to be linked to a higher level of frailty and a greater risk of frailty in this group of older individuals.	The initial measurements of vitamin K levels were conducted, but the observed correlations may have been underestimated due to the exclusion of participants with the lowest levels. The analysis relied on a single biomarker in blood plasma to assess vitamin K concentration.
Malaguarnera et al., 2020[50]	Italy	521	74.2	frailty phenotype	cross-sectional study	carnitine	The average levels of carnitine in the blood of elderly individuals with frailty were lower.	This was a study that involved a cross-sectional approach, so it is important to consider the possibility of reverse causation. Due to ethical considerations, it was not feasible to measure carnitine levels in skeletal muscle tissue through a biopsy.
Ngestiningsih et al., 2021[51]	Bali	27	67.9	Cardiovascular Health Study Scale-based frailty questionnaire	cross-sectional study	IL-6, IL-1β, and vitamin D	The concentration of vitamin D in the body is strongly and consistently linked to the level of frailty in older women.	the sample size was not large;
Pillatt et al., 2021[52]	Brasil	78	75.2	Fried's frailty criteria	Cross-sectional, analytical, and probabilistic study	vitamin D	vitamin D and prediction of gait time	The sample size was not extensive, making it impossible to establish a causal relationship. Additionally, it is important to mention that this research did not consider the presence and quantity of concurrent illnesses that could impact the condition of FS.
Pilleron et al., 2019[53]	France Spain Italy	1324	77.6	Fried's criteria	cross-sectional study	α-,β-carotene, lycopene, cryptoxanthin, lutein, zeaxanthin, retinol, α-,γ-tocopherol, vitamin D3	Individuals with a high carotene intake and low levels of vitamins E and A were more likely to experience frailty.	several criteria of FS were indicated by the patients themselves, which could lead to an underestimation of the number of cases of frailty; The lack of additional indicators that could elucidate the processes underlying the relationship between the discovered structure and vulnerability
Rattray et al., 2019[54]	England	1191	67.7	Fried's criteria	cohort study	metabolic phenotype	The malfunction of the carnitine transport system and the vitamin E pathways contribute to the likelihood of frailty.	a sample of 1,500 cases and 3,500 control groups may not be powerful enough; The instruments employed can be deemed insufficient, as evidenced by the substantial uncertainty surrounding the causal inferences.
Vaes et al., 2019[55]	Netherlands	756	74.0	Fried Frailty Criteria, CES-D questionnaire	cross-sectional study	25-hydroxyvitamin D	The levels of 25(OH)D in the blood were found to be strongly linked to the state of frailty.	The incidence of frailty was comparatively low compared to the reported incidence of physical debility among the elderly population in the area; parathyroid hormone can potentially mediate the relationship
Xiao et al., 2020[56]	China	1324	92.9	Study of Osteoporotic Fractures (SOF) index.	Longitudinal Cohort Study	Plasma 25-hydroxyvitamin D level	The concentration of 25(OH) D was negatively correlated with the likelihood of frailty among the oldest members of the Chinese community.	In this research, the pre-existing condition was not considered; some health issues were self-reported by the participants. This was a descriptive study that did not allow us to determine whether there was a change in the level of 25(OH)D. It is unclear whether this

								change was a cause or a result of the weakening of the body.
**Lipid biomarkers**								
Arauna et al., 2021[57]	Chile	55	72.0	Fried criteria	case-control study	Circulating microvesicles (cMVs)	Frail older adults show a distinct plasma cMVs profile	small sample size
Yin et al., 2023[59]	China	8791	71.9	modified frailty index	cross-sectional study	Cholesterol	The relationship between cholesterol levels and physical frailty in older adults living in the community.	The levels of these biological indicators in the inhabitants of two regions were assessed using distinct instruments, which were restricted to quantifying the relationship between biological indicators and discomfort. No data was gathered regarding the use of medications or the presence of infections during the blood test, which could impact the outcomes of determining biological markers. Additionally, biochemical indicators of bone health were not tested.
**Acid biomarkers**								
Brunelli et al., 2021[60]	Italy	130	77.0	Frailty Index	longitudinal population-based study	hippuric acid<br>43 metabolites	Reduced levels of hippuric acid in the blood as a potential indicator of frailty.	Initially, we were unable to assess the impact of microbial reconfiguration on the low levels of hippuric acid in the elderly participants due to the absence of microbiome data. Furthermore, the dietary habits of the InveCe.Ab elderly participants were not determined using a standardized food frequency questionnaire, but rather through simple inquiries about their weekly food intake.
Jang et al., 2020[61]	South Korea	73	70.8	Fried frailty phenotype and Rockwood frailty index	Cohort study	kynurenine	The concentration of kynurenine in the blood was found to be positively correlated with the frailty index.	The structure of the cross-sectional study does not permit us to establish a causal connection between serum kynurenine levels and FS. The study primarily concentrated on the kynurenine level. The average age of the participants in this study (69.4 years) was considered relatively young for research on aging.
**Metal biomarkers**								
Wei et al., 2022[62]	China	1545	91.8	frailty index	cross-sectional study	blood lead exposure	Elevated blood lead levels may elevate the risk of frailty in the oldest individuals by increasing the likelihood of impairment in four physical abilities.	causal relationship between blood lead content and FS could not be clearly determined; this study was conducted using data on older people in China, so the results may not be applicable to other ages or ethnic groups; the lead content in hair and bones is an important indicator reflecting the level of lead in the body, but in this study these indicators were not measured; they have not classified or studied chronic diseases taking into account their characteristics

Zawadzki et al., 2021[63]	Poland	120	74.6	Edmonton Frailty Scale	cross-sectional study	iron	a decrease in iron concentration was associated with frailty syndrome.	Nevertheless, this investigation did not reveal a significant association between the presence of frailty syndrome and hepcidin levels. The absence of a correlation in this regard may be due to the limited number of participants and the presence of acute inflammatory conditions in patients without frailty syndrome, which could have a substantial impact on the statistical relationship.
**Enzyme biomarkers**								
Sanz et al., 2022[64]	Spain	228	84.9	ADL,Fried Frailty Index, Tilburg Frailty Indicator, Clinical Frailty Scale	RCT	Angiotensin-converting enzyme (ACE) and angiotensin-converting enzyme 2 (ACE2)	Elevated ACE2 activity in the bloodstream was linked to a higher BMI, diminished physical abilities, increased reliance on assistance, and a greater susceptibility to frailty.	the lack of information about the drugs taken by the participants, especially for hypertension; The findings presented herein cannot be generalized to a group that does not meet the requirements for participation in this research.
**Genetic biomarkers**								
Agostini et al., 2023[69]	Italy	186	82.4	Fried index	RCT	microRNAs	The serum miR-451a level should be explored as a potential indicator of frailty.	other factors, including medications, diet, concomitant diseases, etc., could affect microRNA expression
Carini et al., 2022[70]	Italy	41		Fried index	Cohort study	miR-101-3p and miR-142-5p	miR-101-3p and miR-142-5p are significantly reduced in elderly individuals.	the results were obtained on a limited number of subjects; small RNA sequencing was performed on whole blood samples, while the expression levels of some microRNAs in plasma and serum differ; the microRNA target genes discussed here are only bioinformatic predictions
Grasselli et al., 2022[72]	Italy	85	81.9	Fried index	Cohort study	DNA damage in hematopoietic cells	In vulnerable population, we observed elevated oxidative stress and a significant amount of DNA damage in cHPSC.	In the context of a vulnerable population, the clinical profile (age, nutritional status, and medication use) and the presence of comorbidities (diabetes, cardiovascular issues, kidney problems, and respiratory disorders) that contribute to oxidative stress can explain the elevated levels of DNA damage in cHPSC.
Inglés et al., 2019[73]	Spain	152	77.9	Fried criteria	Longitudinal population-based cohort study	DNA using the Chemagic DNA blood kit	Fifteen single-nucleotide polymorphisms, eighteen genes, and four biological pathways, including the interaction between cytokines and their receptors, the cytotoxicity of natural killer cells, the regulation of autophagy, and the renin-angiotensin system, were found to be the most strongly linked to frailty.	none of the genetic variants achieved statistical significance after proper adjustment with Bonferroni correction; relatively small sample size
Iparraguirre et al., 2023[74]	Spain	70	81.0	Fried criteria	cohort study	RNA-Seq profiling	89 differentially expressed circRNAs with frailty.	high heterogeneity that characterizes the frail phenotype<br>the sample size was not large;
Juárez-Cedillo et al., 2019[75]	Mexica	984	70.0	Fried criteria	cohort study	Interleukin 10 gene polymorphisms	The genetic variants rs1800896 and rs1800871 were found to be recessive in their relationship with frailty.	The study was conducted only on the Mexican population.
Lee et al., 2022[76]	Taiwan	189	77.2	Fried Frailty Index	observational study	36 mRNA expression, TNF-α,CXCL10	The expression of CD36 mRNA in peripheral blood mononuclear cells (PBMCs) in healthy older adults is significantly lower compared to those in pre-frail and frail individuals.	the data cannot reflect the actual level of CD36 proteins; could not accurately determine the elevated CD36 mRNA for the corresponding mature receptor-carrying cell type; The findings were assessed at a specific moment in time. This study was a snapshot, and we were unable to establish a causal relationship

								between CD36 mRNA levels and frailty. The sample size was limited, particularly in the frailty group.
Martínez-Ezquerro et al., 2019[77]	Mexico	202	69.9	Fried criteria	cross-sectional study	telomere length	The length of telomeres could potentially serve as a distinguishing factor between healthy and unhealthy aging, as indicated by the frailty phenotype.	even with the initial recruitment of 1,252 people, only 202 participants were included, which meant that recruitment skills were insufficient
Mourtzi et al., 2019[78]	Greece	1234	82.0	Fried definition	retrospective cross-sectional study	Apolipoprotein (APOE) ε4 allele	Following the categorization of APOE genotype, individuals with the APOE ε4 heterozygous variant were found to have a 2.675 times greater likelihood of developing frailty.	did not exclude the possibility that the results obtained are distorted by subclinical stages of dementia; It is conceivable that the impact of apolipoprotein could be influenced by other as yet undiscovered genetic and environmental factors.
Rabaneda-Bueno et al., 2021[79]	Mexica	630	77.0	Fried's criteria	cross-sectional, population-based cohort study	PTPN22 gene functional polymorphism (rs2476601)	The PTPN22 variant, rs2476601, may contribute to frailty and affect the quality of life.	The T variant was less common in the study, with a frequency of approximately 6%.
Selenius et al., 2024[71]	Finland	1605	61.5	frailty index	longitudinal study	brain insulin receptor	The greater diversity in the operation of the insulin receptor gene network in the hippocampus is linked to a growing vulnerability in women.	Approximately one in six participants had passed away by the time of the final measurement, which could potentially weaken the longitudinal connections identified in the study, as they may have been more vulnerable. Furthermore, all the participants were from Helsinki, Finland, which could impact the generalizability and applicability of the findings.
**Urinary biomarkers**								
Jiang et al., 2020[80]	China	230	83.9	Fried criteria	cross-sectional study	8-oxo-7,8-dihydroguanosine (8-oxo-Gsn)	The level of 8-oxo-Gsn in urine was found to be independently linked to frailty. This urinary biomarker could potentially serve as a valuable indicator of frailty.	the causal relationship between oxidative stress and decreased physical activity was not evaluated; circadian variability of cytokines
Liang et al., 2020[81]	China	508	75.0	Fried phenotype	cross-sectional study	Urinary 8-oxo-7,8-dihydroguanosine	The urinary 8-oxoGsn, a well-established indicator of RNA oxidation, is independently linked to frailty in older individuals with cardiovascular issues.	There is no universally accepted method for assessing frailty, although the Fried phenotype, which was employed in this research, is widely recognized as a diagnostic tool. The study is a snapshot in time, so it is not possible to predict the future based on the biomarkers of frailty in elderly individuals with cardiovascular conditions. The elderly patients admitted to the hospital with cardiovascular issues do not fully reflect the broader population of the elderly.
**Salivary biomarkers**								
Furtado et al., 2020[82]	Portugal	358	83.0	Fried's physical frailty components	cross-sectional exploratory study	pro and anti-inflammatory cytokines, sex steroid hormones, salivary anti-microbial proteins, blood cells counts	Salivary α-amylase was the most effective indicator of frailty, as it correlated with all five aspects of physical frailty.	The number of participants was comparatively low, considering the individual differences in some of the biomarkers analyzed; cross-sectional data do not allow to establish a causal relationship
Gómez-Rubio et al., 2022[83]	Spain	72	83.3	frailty score	cross-sectional pilot study	Salivary IL-6	The level of IL-6 in saliva can serve as a potential indicator of frailty and a means to assess the impact of interventions on frail individuals.	small sample size; das not exclude the influence of all concomitant factors that can change the level of IL-6

S3. Reporting checklist for systematic review (with or without a meta-analysis).

Based on the PRISMA guidelines.

Instructions to authors

Complete this checklist by entering the page numbers from your manuscript where readers will find each of the items listed below.

Your article may not currently address all the items on the checklist. Please modify your text to include the missing information. If you are certain that an item does not apply, please write "n/a" and provide a short explanation.

Upload your completed checklist as an extra file when you submit to a journal.

In your methods section, say that you used the PRISMAreporting guidelines, and cite them as:

Page MJ, McKenzie JE, Bossuyt PM, Boutron I, Hoffmann TC, Mulrow CD, Shamseer L, Tetzlaff JM, Akl EA, Brennan SE, Chou R, Glanville J, Grimshaw JM, Hróbjartsson A, Lalu MM, Li T, Loder EW, Mayo-Wilson E, McDonald S, McGuinness LA, Stewart LA, Thomas J, Tricco AC, Welch VA, Whiting P, Moher D. The PRISMA 2020 statement: An updated guideline for reporting systematic reviews

		Reporting Item	Page Number
**Title**			
Title	#1	Identify the report as a systematic review	1
**Abstract**			
Abstract	#2	Report an abstract addressing each item in the PRISMA 2020 for Abstracts checklist	1
**Introduction**			
Background/rationale	#3	Describe the rationale for the review in the context of existing knowledge	1-3
Objectives	#4	Provide an explicit statement of the objective(s) or question(s) the review addresses	3
**Methods**			

Eligibility criteria	#5	Specify the inclusion and exclusion criteria for the review and how studies were grouped for the syntheses	4
Information sources	#6	Specify all databases, registers, websites, organisations, reference lists, and other sources searched or consulted to identify studies. Specify the date when each source was last searched or consulted	4
Search strategy	#7	Present the full search strategies for all databases, registers, and websites, including any filters and limits used	3
Selection process	#8	Specify the methods used to decide whether a study met the inclusion criteria of the review, including how many reviewers screened each record and each report retrieved, whether they worked independently, and, if applicable, details of automation tools used in the process	3-4
Data collection process	#9	Specify the methods used to collect data from reports, including how many reviewers collected data from each report, whether they worked independently, any processes for obtaining or confirming data from study investigators, and, if applicable, details of automation tools used in the process	4
Data items	#10a	List and define all outcomes for which data were sought. Specify whether all results that were compatible with each outcome domain in each study were sought (for example, for all measures, time points, analyses), and, if not, the methods used to decide which results to collect	4
Study risk of bias assessment	#11	Specify the methods used to assess risk of bias in the included studies, including details of the tool(s) used, how many reviewers assessed each study and whether they worked independently, and, if applicable, details of automation tools used in the process	5
Effect measures	#12	Specify for each outcome the effect measure(s) (such as risk ratio, mean difference) used in the synthesis or presentation of results	5
Synthesis methods	#13a	Describe the processes used to decide which studies were eligible for each synthesis (such as tabulating the	5

		study intervention characteristics and comparing against the planned groups for each synthesis (item #5))	
Synthesis methods	#13b	Describe any methods required to prepare the data for presentation or synthesis, such as handling of missing summary statistics or data conversions	5
Synthesis methods	#13c	Describe any methods used to tabulate or visually display results of individual studies and syntheses	5
Synthesis methods	#13d	Describe any methods used to synthesise results and provide a rationale for the choice(s). If meta-analysis was performed, describe the model(s), method(s) to identify the presence and extent of statistical heterogeneity, and software package(s) used	5
Synthesis methods	#13e	Describe any methods used to explore possible causes of heterogeneity among study results (such as subgroup analysis, meta-regression)	5
Synthesis methods	#13f	Describe any sensitivity analyses conducted to assess robustness of the synthesised results	5
Reporting bias assessment	#14	Describe any methods used to assess risk of bias due to missing results in a synthesis (arising from reporting biases)	5
Certainty assessment	#15	Describe any methods used to assess certainty (or confidence) in the body of evidence for an outcome	5
Data items	#10b	List and define all other variables for which data were sought (such as participant and intervention characteristics, funding sources). Describe any assumptions made about any missing or unclear information	4
**Results**			
Study selection	#16a	Describe the results of the search and selection process, from the number of records identified in the search to the number of studies included in the review, ideally using a flow diagram (http://www.prisma-statement.org/PRISMAStatement/FlowDiagram)	5

Study selection	#16b	Cite studies that might appear to meet the inclusion criteria, but which were excluded, and explain why they were excluded	6
Study characteristics	#17	Cite each included study and present its characteristics	6
Risk of bias in studies	#18	Present assessments of risk of bias for each included study	8
Results of individual studies	#19	For all outcomes, present for each study (a) summary statistics for each group (where appropriate) and (b) an effect estimate and its precision (such as confidence/credible interval), ideally using structured tables or plots	7-8
Results of syntheses	#20a	For each synthesis, briefly summarise the characteristics and risk of bias among contributing studies	7-8
Results of syntheses	#20b	Present results of all statistical syntheses conducted. If meta-analysis was done, present for each the summary estimate and its precision (such as confidence/credible interval) and measures of statistical heterogeneity. If comparing groups, describe the direction of the effect	8
Results of syntheses	#20c	Present results of all investigations of possible causes of heterogeneity among study results	8
Results of syntheses	#20d	Present results of all sensitivity analyses conducted to assess the robustness of the synthesised results	8
Risk of reporting biases in syntheses	#21	Present assessments of risk of bias due to missing results (arising from reporting biases) for each synthesis assessed	8
Certainty of evidence	#22	Present assessments of certainty (or confidence) in the body of evidence for each outcome assessed	8
**Discussion**			
Results in context	#23a	Provide a general interpretation of the results in the context of other evidence	9-11
Limitations of included studies	#23b	Discuss any limitations of the evidence included in the review	11

Limitations of the review methods	#23c	Discuss any limitations of the review processes used	11
Implications	#23d	Discuss implications of the results for practice, policy, and future research	12
**Other information**			
Registration and protocol	#24a	Provide registration information for the review, including register name and registration number, or state that the review was not registered	12
Registration and protocol	#24b	Indicate where the review protocol can be accessed, or state that a protocol was not prepared	12
Registration and protocol	#24c	Describe and explain any amendments to information provided at registration or in the protocol	12
Support	#25	Describe sources of financial or non-financial support for the review, and the role of the funders or sponsors in the review	12
Competing interests	#26	Declare any competing interests of review authors	12
Availability of data, code, and other materials	#27	Report which of the following are publicly available and where they can be found: template data collection forms; data extracted from included studies; data used for all analyses; analytic code; any other materials used in the review	12

The PRISMA checklist is distributed under the terms of the Creative Commons Attribution License CC-BY. This checklist was completed on 19. February 2025 using https://www.goodreports.org/, a tool made by the EQUATOR Network in collaboration with Penelope.ai
